# Using Genealogical Mapping and Genetic Neighborhood Sizes to Quantify Dispersal Distances in the Neotropical Passerine, the Black-Capped Vireo

**DOI:** 10.1371/journal.pone.0140115

**Published:** 2015-10-13

**Authors:** Giridhar Athrey, Richard F. Lance, Paul L. Leberg

**Affiliations:** 1 Department of Poultry Science, Texas A&M University, 2472 TAMU, College Station, Texas, United States of America; 2 Faculty of Ecology and Evolutionary Biology, Texas A&M University, College Station, Texas, United States of America; 3 Department of Biology, University of Louisiana at Lafayette, P.O. Box 42451, Lafayette, LA, United States of America; 4 Environmental Laboratory, USACE, Vicksburg, MS, United States of America; Liverpool John Moores University, UNITED KINGDOM

## Abstract

Dispersal is a key demographic process, ultimately responsible for genetic connectivity among populations. Despite its importance, quantifying dispersal within and between populations has proven difficult for many taxa. Even in passerines, which are among the most intensely studied, individual movement and its relation to gene flow remains poorly understood. In this study we used two parallel genetic approaches to quantify natal dispersal distances in a Neotropical migratory passerine, the black-capped vireo. First, we employed a strategy of sampling evenly across the landscape coupled with parentage assignment to map the genealogical relationships of individuals across the landscape, and estimate dispersal distances; next, we calculated Wright’s neighborhood size to estimate gene dispersal distances. We found that a high percentage of captured individuals were assigned at short distances within the natal population, and males were assigned to the natal population more often than females, confirming sex-biased dispersal. Parentage-based dispersal estimates averaged 2400m, whereas gene dispersal estimates indicated dispersal distances ranging from 1600–4200 m. Our study was successful in quantifying natal dispersal distances, linking individual movement to gene dispersal distances, while also providing a detailed look into the dispersal biology of Neotropical passerines. The high-resolution information was obtained with much reduced effort (sampling only 20% of breeding population) compared to mark-resight approaches, demonstrating the potential applicability of parentage-based approaches for quantifying dispersal in other vagile passerine species.

## Introduction

Gene flow within and among populations is one of the essential attributes of genetic connectivity across space. However, genetic connectivity does not necessarily translate to demographic connectivity, which is necessary for population stability [1, 2]. Demographic connectivity depends on measurable population growth that can be due to immigration, whereas genetic connectivity helps us understand the consequences of dispersal over evolutionary time [1]. Estimates of gene flow can thus indicate genetic connectivity, without shedding light on the processes maintaining demographic connectivity. Dispersal is one of the main population processes that drive both demographic connectivity, and in turn genetic connectivity. Dispersal in vertebrate species has been defined as the permanent, unidirectional movement of individuals away from their site of birth to the site of reproduction (Greenwood and Harvey 1982). As population genetic structure is a direct consequence of dispersal patterns, quantifying the relationship between dispersal and gene-flow in natural populations requires precise estimates of dispersal rates and distances [3]. Despite the central role played by dispersal, the relationship between individual movement and gene flow is not well characterized. Quantifying dispersal and understanding its relation to breeding biology, social structure and gene flow can be considered among of the most important problems in evolutionary ecology. In most species, dispersal behaviors directly influence patterns of gene flow and population structure [4, 5].

Quantifying the number of dispersers can be logistically challenging with direct observational methods [4, 6], typically requiring time- and resource-intensive studies. This difficulty in quantifying dispersal is further compounded by the disparity between dispersal capability and observed rates of dispersal in several organisms [5, 7]. Furthermore, even when estimates of gene flow can be inferred (for example, using *F*_ST,_ a measure of within and between population partitioning of genetic variation), measures of high genetic-connectivity may not indicate demographic connectivity and, hence, interdependence [1]. Furthermore, *F*_*ST*_-based estimates make several assumptions that are often violated, such as infinite population size or random mating, and can misinform the inferences on genetic connectivity[8]. Thus measures of individual movement and local demographic process can provide better context for gene flow estimates, especially when genetic and demographic connectivity is a concern, as in the cases of conservation or management of threatened and endangered species.

Other than intrinsic demographic processes promoting movement, dispersal behaviors and rates can also be influenced by extrinsic (and anthropogenic) factors, including habitat loss, fragmentation and climate change [9, 10]. For vulnerable or endangered populations, lack of dispersal, and isolation can potentially lead to accumulation of inbreeding, reduced fitness and declines in genetic diversity [11]. Although the long-term genetic consequences of fragmentation are to a large extent unknown, evidence suggests increases in genetic structure with habitat fragmentation [12–14], and that change in population genetic structure may precede losses of diversity in fragmented populations [9]. Better knowledge of dispersal rates, distances and the factors that influence individual movement will be valuable in the design and maintenance of important habitats and movement corridors [15], and ultimately, maintenance of genetic diversity.

Historically, dispersal has been inferred from direct observational methods, such as capture-mark-recapture (CMR) methods, but among the oft-cited pitfalls of these methods are the inability to separate mortality from dispersal and the biases arising from failure to record long distance and pre-capture dispersal [16, 17]. Ideally, dispersal would result in gene flow within and between populations; however this is seldom the case, as there is usually a disparity between measures of the within and between population gene flow. Indirect estimators based on Wright’s *F*-statistics, and population assignment methods [18] have become surrogate measures of inter-population gene flow, and by extension–of dispersal. Due to the various assumptions made by *F*_*ST*_ based estimators, their suitability for the estimation of migrants is limited [19, 20]. Currently available population assignment approaches are also limited in that they do not offer resolution of individual movements within populations that lack subdivision [6].

In the absence of population subdivision, dispersal rates and distances can be inferred using both individual and population level estimates. Parentage analysis and assignment is an individual based approach that is increasingly used to answer questions regarding mating systems [21], detect pre-capture dispersal [16]. More recently, parentage analysis has also become a viable approach to estimate dispersal distances [22, 23], provided appropriate sampling. Our strategy was to analyze the parent-offspring relationships from our sampling, and to calculate the distances between each parent and offspring pair, used to generate a distribution of natal dispersal distances. This approach provides the most direct estimate of dispersal distance, comparable to, and in some instances better than, direct capture-mark-recapture methods [16]

A complementary, inter-population approach is to use sampling based on fine-scale population data to estimate the effective number of dispersers [24, 25], and describe the genetic neighborhood size. As gene dispersal estimates are a function of effective density of the population, they provide a measure of the dispersal events that translated into reproductive success (successful movement of alleles across space) over time. This measure, however, could also refer to the inter-patch dispersal rate, as these estimates are unaffected by rare long-distance immigrants [26]. It is important to note, however, that even with these methods, long-distance dispersal distances cannot be quantified, as this requires sampling multiple populations, which was not in the scope of this study.

The black-capped vireo (*Vireo atricapilla*) is a Neotropical passerine that breeds in the mid-seral oak-juniper savanna of central Texas and southern Oklahoma [27]. While its historical range is reported to have been extensive, habitat loss has reduced the current extent of suitable habitat by up to 50% [28]. In prior studies on this species, we reported that despite high genetic variability, contemporary populations have experienced a substantial loss of genetic diversity, while also becoming increasing differentiated [14]. This is an intriguing result considering the vagility of this migratory species. We also reported sex-biased dispersal in black-capped vireos, indicated by the significant spatial autocorrelation of male relatedness at short distances, compared to that for females [29]. However, an important component of its life history that is not clearly understood concerns the rate and scale of dispersal that can translate into gene flow across the scale of the available habitat. The species is currently listed as ‘Vulnerable’ by the IUCN, but was listed as ‘Endangered’ at the time of sampling (2005–2009). Our main objective in the present study was to characterize natal dispersal within a population of this species. We estimated these aspects of dispersal by using two complementary genetic approaches, based on species-specific microsatellite markers. Our study addresses an important uncertainty in the knowledge about dispersal in migratory passerines, which is crucial for understanding evolution of dispersal behaviors and for the development of conservation strategies for habitat-sensitive species.

## Materials and Methods

### Ethics statement

At the time of the study, the U.S. Fish & Wildlife Service listed the black-capped vireo as a threatened species. All field sampling was conducted based on the guidelines of the U.S. Geological Survey Bird Banding Laboratory (BBL). The master permit for threatened/endangered species included explicit information on and number and locations of individuals to be captured, and these limits were strictly adhered to. All captures were recorded and reported at the end of each banding season to the BBL. All sampling in this study was carried out on protected lands.

Location of study and sample collection

**Fig 1:**
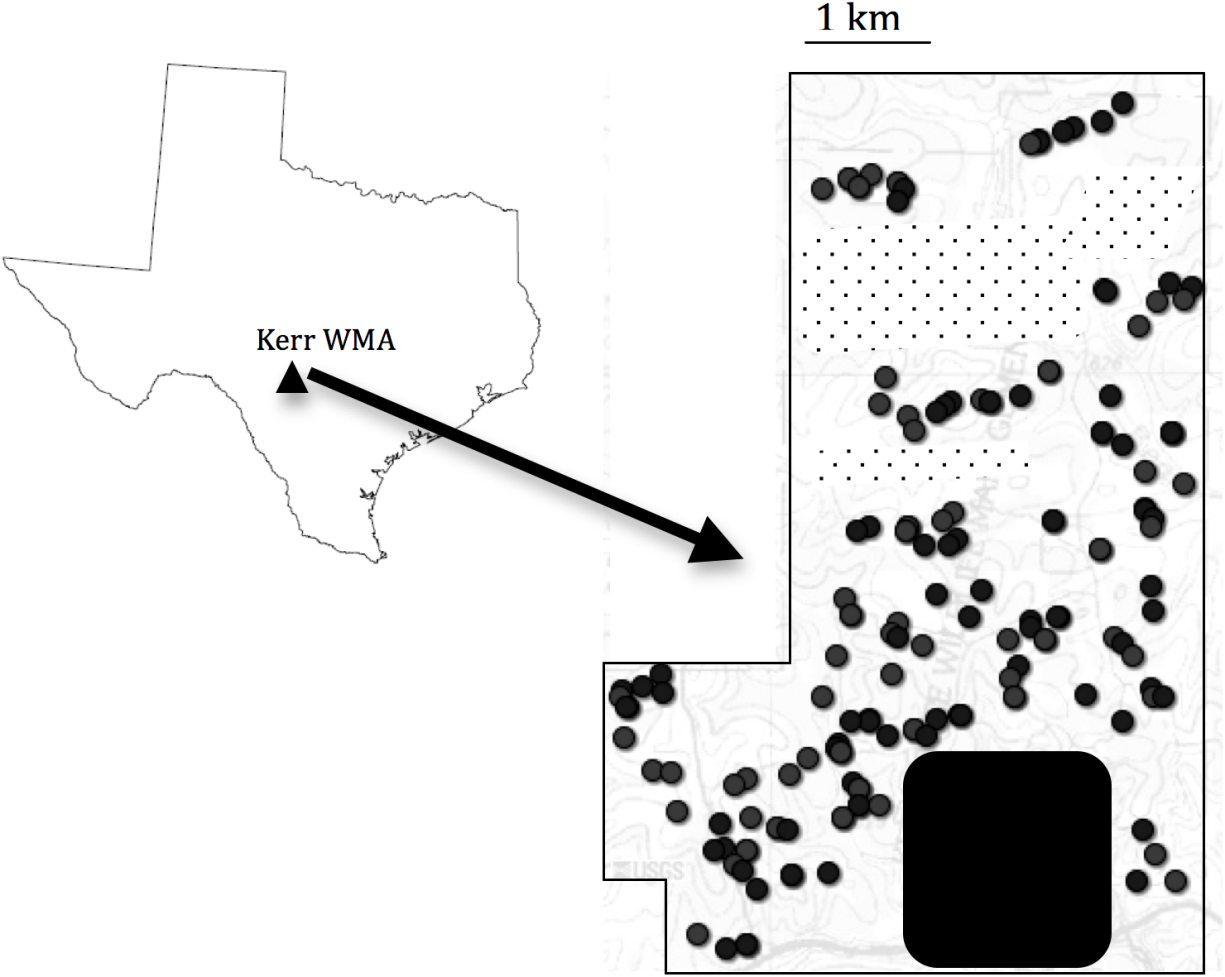
Map of the state of Texas, with KWMA enlarged. The image shows the sampling locations at KWMA. Filled circles denote the sample locations of birds for a total of 160 sampled individuals. The dot-patterned and the black shading shows parts of KWMA that is considered unsuitable habitat for Black-capped vireos. The suitable habitat portion of KWMA is estimated to be 24^2^ km.

Our study was conducted during the breeding seasons in 2007 and 2008, at the Kerr Wildlife Management Area (KWMA), Kerr County, TX (Fig 1). The breeding season stretches from March through August, but most territory defending and mating occurs in the early part, with males and females becoming less responsive after July, when they are occupied in raising chicks. All our sampling in this study was completed by July 1^st^. Eighty individuals were sampled in each year for a total of 160 individuals (annual sampling limits imposed by USFWS permits). Individual birds were captured using male song playback, alarm call (referred to as a shrad call), or rarely using screech owl (*Otus asio*) calls with a speaker placed on each side of a mist-net. Territorial males, and often, females were captured. Upon capture, a U.S. Fish and Wildlife Service (USFWS) identifying band was placed on each bird, and 5–20μl of venal blood was obtained by venipuncture. Blood was immediately stored in 600μl cell-lysis solution and refrigerated until extraction of DNA. We used a Qiagen-Purgene DNA extraction protocol (Qiagen, Valencia, CA) from non-mammalian whole blood. Isolated DNA was eluted in 100μl nuclease-free Hydration Solution, until further processing for genotyping. Individual DNA samples were genotyped at twelve microsatellite loci that were all designed for this species [30].

### Sampling design

The KWMA encompasses approximately 40 km^2^ area, ranging from grassland habitat to oak-juniper scrub to old-growth juniper monoculture. About 60% (24 km^2^) of the KWMA was habitat suitable for black-capped vireo (at the time of sampling). The suitable habitat areas were divided into forty 1 km^2^ grids and approximately the same numbers of birds (three) were sampled in each grid to ensure that the entire area of suitable vireo habitat was sampled equally. If the capture locations of individuals are not the same as their breeding territories, then distance estimates can be imprecise. In this study, the locations of most breeding males and their nest sites were known due to ongoing nest surveys (source: KWMA). As a consequence of this knowledge, our captures were targeted at known individual territories. Secondly, while it is possible that particular BCVI males traveled across other territories to respond to male playback, breeding males are extremely aggressive to intruding males, and are seldom observed on territories other than their own ([27], G. Athrey, personal observation).

### DNA extraction and genotyping

DNA extraction and genotyping was carried out using the same protocol as described in Barr *et al*. [31]. As parentage assignment is highly sensitive to genotyping errors, we followed the recommendations of Bonin [32] to minimize error rates in our amplification and genotyping protocol. Individual loci were amplified and genotyped three times each, and a genotype was considered reliable if observed at least two times out of the three replicates. We used the program MICROCHECKER [33] to check for the presence of null alleles in the dataset, and the program GIMLET [34] to estimate error rates and obtain a consensus dataset. Allelic data was tested for deviations from Hardy-Weinberg equilibrium (HWE) on the online portal GENEPOP [35]. We estimated expected heterozygosity (*H*_*EXP*_) in the program GENETIX [36].

Parentage assignment estimation of natal dispersal

### Parentage analysis

We used a categorical likelihood approach to assign individuals to candidate parents in the program CERVUS Version 3.03, [37]. All males and females in the sample (excluding the focal individual) are equally likely to be a parent, and this hypothesis is evaluated by a likelihood approach. In contrast to the exclusion or fractional likelihood approaches, the categorical assignment approach is more biologically intuitive as it assigns whole offspring genotypes to candidate parents [38]. Additionally, the categorical likelihood method does not require an *a priori* specification of the probabilities of parentage. This was more appropriate than the fractional likelihood approach in our case as there is little data upon which such a prior probability can be defined. The categorical allocation method selects the most likely parent from a pool of candidate parents by calculating the logarithm of the odds ratio (LOD score) for each candidate. Among the candidate parents, the individual with the highest LOD score is assigned as the parent. When LOD scores are zero, offspring are not assigned to any parents. This method is also reported to be forgiving of small amounts error arising out of genotyping protocols or mutations

We performed the categorical parentage assignment on our dataset of 160 individuals. Parentage assignment was preceded by simulations to assess the significance of paternity inference based on the allele frequencies in the sampled population. One hundred thousand simulation runs were used to assess confidence in the assignments. The simulation approach generates populations of parents and offspring, together with a user-specified genotyping error rate. Assignments are calculated based on the likelihood of odds ratio (LOD score), and a statistic ∆ is calculated for each assignment. The simulation approach generates a distribution of ∆ values comparing the correct to the false assignments from the simulated data. This approach helps determine the critical ∆ value for confidence in assignment from empirical data. For each offspring-candidate parent pair, confidence of assignment is evaluated relative to the simulated threshold. This approach is considered one of the most robust methods of parentage assignment currently available [39] [40] as it allows the estimation of confidence for each parent-offspring assignment, but also an experiment-wide error rate [38, 41]. Depending on the LOD score, the confidence of individual parent-offspring assignments can be higher or lower than 95%, and is considered a population average. At 80% or ‘relaxed’ confidence, the lower probability of assignment takes into account possible mismatches due to genotyping errors or mutations, and the relaxed criterion provides estimates with a lower certainty threshold [37, 42]. Genotyping errors are the major source of errors in parentage analysis and in this study we used a variety of approaches to reduce this source of error (details presented above).

Another concern with parentage assignments is the potential inability to distinguish between father-offspring versus full-sib brother assignments. In our analysis, parentage was assigned by using available age data for the sampled individual. Aging of individuals in the field can be imperfect, especially in after-second year birds (ASY). However, females, after hatch years (AHY), second year (SY) and after-second year (ASY) can be clearly distinguished. The time of our sampling excluded sampling of hatch year (HY) birds.

The purpose of paternity analysis is two fold: in one situation, the number of paternities assigned is the most important information [43], whereas in other cases, the accuracy of paternity assignment is crucial [37]. Each of these necessities of parentage analysis is met by the relaxed and strict assignments, respectively. However, for the purposes of distance calculation, the total number of paternities assigned is more informative. Hence we included the assignments at the relaxed confidence level in the calculations of dispersal distances. Moreover, the proportions of individuals dispersing different distances were not significantly different for strict and relaxed confidence measures. Paternity and maternity analyses were performed independently on the dataset. One hundred and sixteen (116) males were included as candidate fathers and 44 females as candidate mothers. Following the paternity and maternity analyses we also analyzed paternity with a known mother (*post hoc*, based on assignment) and vice versa. These *post hoc* analyses were performed to determine if and how the assignment proportions change when a known parent is included.

### Estimation of natal dispersal

The main assumption in the estimation of natal dispersal is that captured individuals have fledged and occupied their breeding territories. In paternity analysis, when the same pool contains both candidate sons and fathers, a father-offspring pair can be reciprocally assigned–based on the LOD score. In such circumstances, the age of the candidate individual was used to assign the father. We felt confident in our assumption for detecting natal dispersal given that 98% of captured males were adults (SY and ASY) that were actively defending a territory. [37]. All captured females were also adults (SY and ASY) and very likely to have completed their post-natal dispersal. We generated a distribution of dispersal distances following assignment of offspring to candidate fathers or mothers.

To determine if similar proportions of males and females were assigned to parents in the study population, relative to those that could not be assigned parents, we used a Fishers exact test. For each pair of assigned offspring-parent, we calculated the Euclidean distance between them. Distances obtained after considering all the assignments were analyzed as one dataset, as well as separately for males and females. We tested whether i) dispersal distances deviated from the null expectation (distribution of unrelated individuals), and ii) male and female dispersal distances were different, using a Mann-Whitney U Test (PROC NPAR1WAY, SAS V9, Cary, NC). To characterize the shape of the distribution of dispersal we first divided the sampled landscape into 500m distance classes and obtained counts for the number of dispersers in each distance class. We divided the sampled habitat into 14 distance classes of 500m each. We chose 500m distance classes based on sample size considerations; if dispersal distances had a relatively uniform distribution, each 500m class would contain at least five individuals. We then tested whether the distributions of male and female dispersal distances were the same using a Kolmogorov-Smirnov test (PROC NPAR1WAY, SAS V9, Cary, NC) [44].

Estimates of long distance movements are typically underestimated when the study area is finite or when sampling is inadequate. In this study we were specifically interested in understanding natal dispersal distances at the subpopulation level as evidence from other studies suggest that dispersal is restricted in black-capped vireos [29]. Second, our sampling protocol was sufficient to sample 20% of all individuals in the population, including 29% of breeding males (based on a known census size of 400 singing males at time of study). While these numbers are not exhaustive, these numbers are similar to or greater than those typically found in genetic studies of vertebrates (eg. [41, 45]. Hence we did not expect insufficient sampling to be a factor in our estimates of dispersal distances. The study population (KWMA) is about 112 km distant from the nearest neighboring population (Rancho Diana, near San Antonio, TX), and given the extent of differentiation between KWMA and Rancho Diana [31], we suspect that long distance natal dispersal is not frequent in this species. Furthermore, the longest recorded dispersal movement based on banding data for the species is 78 km [46].

To compare the distributions of male and female distances to a null expectation and to determine if the limited size of our sample area would influence our distributions of dispersal distances, we looked at the average distances separating unrelated individuals. Unrelated individuals were the group of individuals that were not assigned to any parent-offspring pairs by assignment test. From this group we randomly sampled 100 individuals (with replacement) to generate a distribution of distances. This approach generates a distribution of distances that are presumably not driven by their relationship to other individuals in the population. Hence the distance estimates serve as a null distribution for randomly spaced individuals across the habitat matrix, in contrast to a distribution generated by intentional dispersal. This distribution was compared to the distribution of distances obtained from assigned pairs (above) using a Komogorov-Smirnov test (PROC NPAR1WAY, SAS V9, Cary, NC) [44]. Additionally we calculated the cumulative inverse proportion of disperser counts into each distance class (spaced at 500m intervals) for males and females. Then we determined the fit of this distribution to the exponential decay function and also to determine if the decay was similar for males and females.

Indirect estimates of gene dispersal distance parameters

### Neighborhood size

With the availability of multilocus marker data from intensively sampled populations, the calculation of dispersal distance has become a very useful approach to estimate gene flow and effective dispersal [47, 48]. Using Wright’s equation for neighborhood size for non-selfing diploids, *N*_*b*_ = 4π*D*_*e*_σ^2^, the mean squared distance between the locations of parents and their offspring (σ) can be estimated based on the neighborhood size (*N*_*b*_), and the effective density (*D*_*e*_). This parameter σ is not in itself a measure of dispersal distance. First we calculated the neighborhood size *N*_*b*,_. using on the equation *N*_*b*_ = -(1-*F*)/*b*_*log*_,[49, 50] where *F* is Wright’s inbreeding coefficient. In this instance, we replaced *F* by the mean kinship coefficient, following option #3 in the SPAGedi manual (Inference of Gene Dispersal Distances)[51]. And *b*_*log*_ is the regression slope based upon the pairwise kinship coefficients for all pairs of individuals, on the logarithm of spatial distance. The kinship coefficient is reported to be more informative about population parameters when sampling may include a high proportion of related individuals [51]. For this reason, we chose to use the kinship coefficient [52] instead of Wright’s *F*. We used the program SPAGeDi [51] to estimate the kinship coefficients and the slope of its regression against spatial distances *b*_*log*_. Kinship estimates were sampled by 10000 random permutations to test for significance. Estimates of the kinship coefficients and *b*_*log*_ were used to determine *N*_*b*._ The effective density *D*_*e*_ is defined as *N*_*e*_/study area, where *N*_*e*_ is the effective population size. We estimated *N*_*e*_ using three different estimators (described below), to evaluate the impact of different genetic diversity components on the effective size, and hence on effective dispersal.

*N*_*e*_ based on sibship assignment (SANE): We used the estimator of Wang [53] for single samples based on multilocus genotypes. This approach estimates *N*_*e*_ as a function of the frequencies of full and half-sib dyads in the sample population. We believe that this method is especially appropriate for our study as it accommodates a flexible sampling protocol where samples from two consecutive generations can be sampled at once, as long as the individuals are sampled randomly. Since the method is based on sibship and parentage analysis it requires separation of candidate offspring and parents. We estimated the SANE in the program COLONY2 [53].

*N*_*e*_ based on Linkage Disequilibrium (LDNE): linkage disequilibrium (LD) method is also a single sample estimator of *N*_*e*_ based on estimates of nonrandom association of alleles at different loci in the sample [54]. In closed populations of constant size, the extent of LD is a function of the effective size of the population and the recombination rate, and hence measures of linkage disequilibrium in the sample can be used to estimate *N*_*e*_. This approach is considered to be reliable when large sample sizes are available [55], and we felt that the availability of large samples sizes makes this an informative comparison. We estimated the LDNE using the method of Waples and Do [56].

*N*_*e*_ based on temporal variance (TNE): In contrast to the other two methods the temporal estimator requires at least two samples separated in time by one or more generations [57]. The temporal method is currently the most widely used and reported estimator of short term *N*_*e*_, but is usually limited in its application due to its requirement of samples separated by one or more generations. We used the temporal estimate that we obtained using older samples collected for previous studies [14, 31] to obtain a temporal estimate for the period 2005–08. We estimated TNE using the program MLNE [58, 59].

*D*_*e*_ was estimated for the 24 km^2^ that constitutes the portion of the study area that can be considered suitable habitat for black-capped vireos. Additionally there is no suitable habitat surrounding this population, with only a few territories located immediately on the site’s periphery. The nearest concentration of vireos is approximately 110 km away. Thus, we feel that using the area measure represented by available vireo habitat is the most appropriate for estimating the effective density of individuals in the population.

## Results

We had 116 males and 44 females (total 160) in our dataset. Analysis of our data in GIMLET showed that our average error rate per locus was 0.1%. MICROCHECKER showed no evidence for null alleles in our data. Population genetic estimates for KWMA did not show significant deviations from HWE at any of the 12 loci analyzed. The average heterozygosity across 160 samples was 0.81 (SE = 0.0013).

### Parentage assignment

Paternity analysis of the 159 candidate offspring (1putative parent to 159 candidate offspring out of 160 total) produced 58 assignments at the strict confidence level (fewer than expected based on simulation). Thirteen percent of assigned offspring were females, with the remaining assignments being male offspring (87%). At the relaxed confidence level, the number of offspring assigned increased to 109, of which 82% were male and 18% were female–greater than expected based on simulations (Table 1).

**Table 1:** Summary of parentage assignment estimates. Results of paternity and maternity analysis are listed, along with the expected estimates at two confidence levels. Results include analysis where parents were unknown as well as those where a known parent was specified.

		**Paternity (Mother unknown)**		**Maternity (Father Unknown)**	
		**Assigned**	**Expected**	**Assigned**	**Expected**
	Strict (95%)	58	74	4	17
Confidence level	Relaxed (80%)	109	99	10	21
		**Paternity (Mother known)**		**Maternity (Father known)**	
		**Assigned**	**Expected**	**Assigned**	**Expected**
	Strict (95%)	59	74	9	17
Confidence level	Relaxed (80%)	103	99	14	21

There were fewer assignments to mothers than to fathers. The low number of maternity assignments is the result of our reduced ability to sample females because, territorial males were more likely to respond to playback. At the strict confidence level only four offspring were assigned to mothers, where as at the relaxed confidence level, the number of assignments increased to 10. A total 92% of individuals assigned to a mother were male. When assigned fathers were included in the dataset as a ‘known father’, the number of assignments at the strict confidence level changed to 9 at strict confidence and to 14 at relaxed confidence (Table 1).

Of the 58 assignments at strict confidence level, 44 males explained the paternity of 58 offspring. At the relaxed confidence level, 74 males explained the paternity of 109 individuals. Among the 109 assignments there was a subset of 46 individuals that were assigned as fathers to other individuals, indicating that at least three generations were represented in the sampled population. Twenty-three males had two or more offspring attributed to them, whereas the maximum number of offspring attributed to a single male being was four. Due to the variance in reproductive success suggested by the assignments, we determined that 31% of fathers accounted for 58% of the offspring, with an average of 1.4 offspring per male parent.

### Inferred rate of dispersal from parentage assignment

Based on assignment at the strict or relaxed confidence levels, the proportion of individuals that were successfully assigned parentage within the natal population was as high as 64% (109/159 based on relaxed criteria). These assignment rates suggest that dispersers from outside the study area parented up to 36% of individuals in the population.

In the relaxed criterion, a mismatch at one locus due to genotyping error was allowed in assigning parentage. What this means is that if more than one parent cannot be excluded as being the correct father, one of them is identified as the most likely parent, which comes at lower confidence. At the relaxed confidence interval, 77% of males and 44% of females were assigned to a parent in the natal population, suggesting more females may have originated from outside the study population. The odds of a male being assigned to a parent in the sample population were 4.5 (95% CI = 2.1–9.7) times higher than the odds of assigning a female. Assuming we were not less likely to sample the parents of females than males born in the study population, this result suggests that females were more likely to have immigrated from outside our study area.

### Distribution of dispersal distances

**Fig 2:**
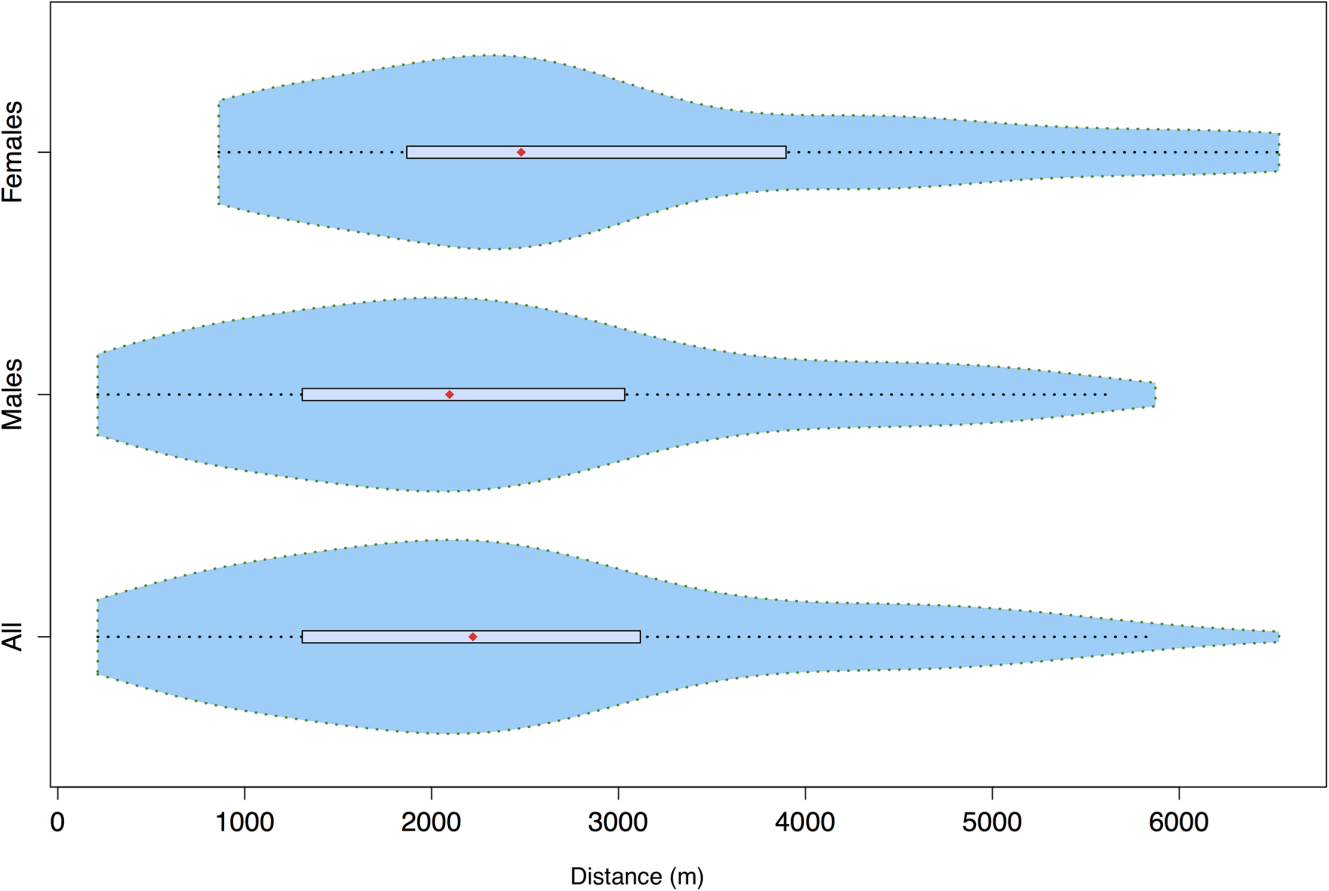
Horizontal violin plots, showing the density distribution of dispersal distances for a) all assigned individuals, b) assigned males and c) assigned females. The box within the plot bounds the interquartile range, whereas the red point shows the location of the median. The x-axis shows the distance in meters that span the sampled landscape.

The median natal dispersal distance for assigned individuals was 2220m, and the distributions were positively skewed for males, females and the combined data (skewness = 1.37, kurtosis = 1.62, Fig 2).

**Fig 3:**
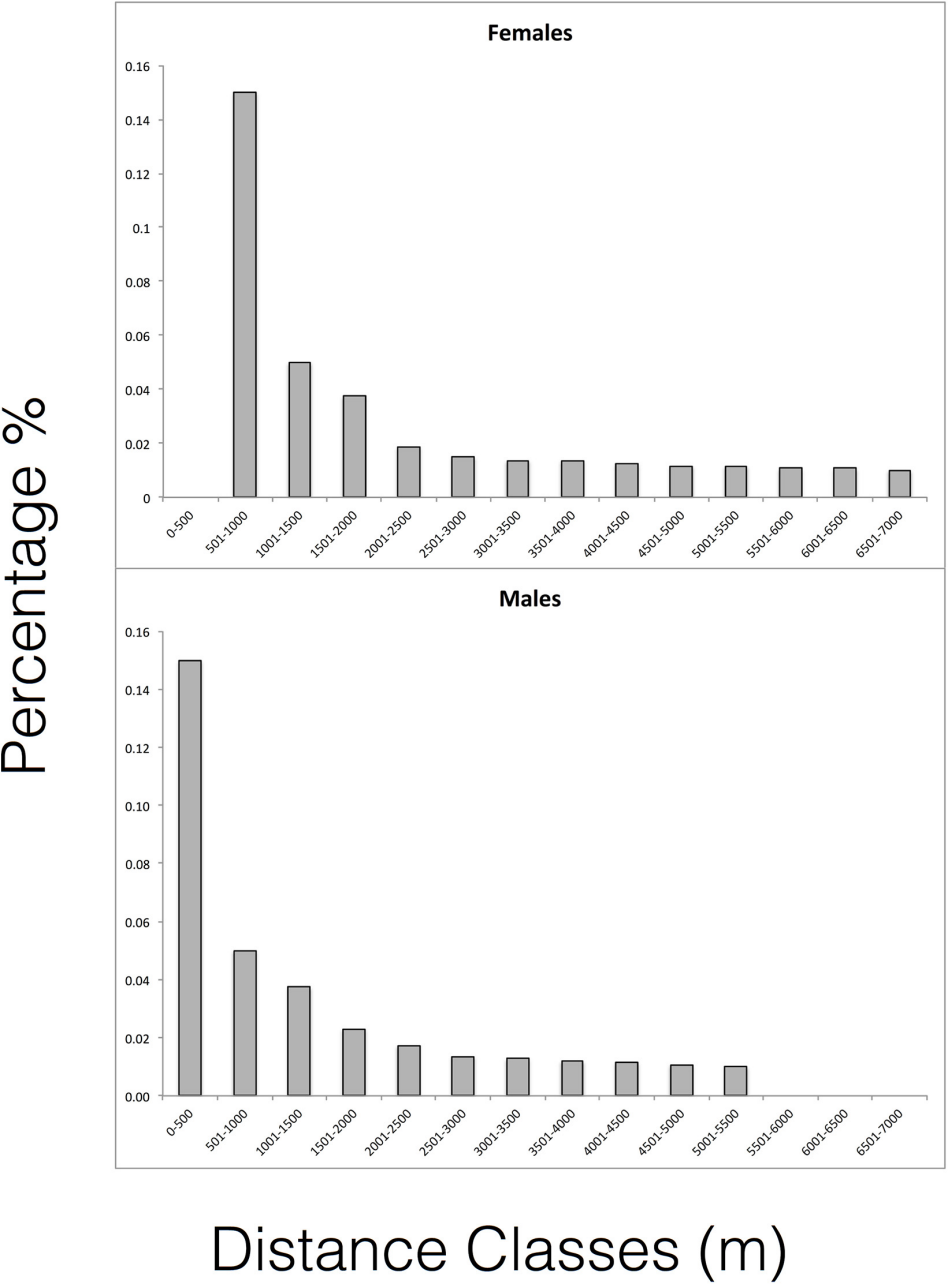
Plots showing the inverse cumulative proportions that represent the decay function fitted to the a) female and b) male dispersal distances. The x-axis shows the distance in meters that span the sampled landscape, divided into 500m distance classes. The y-axis represents the proportion. The negative inverse cumulative distribution of distances for females start at the second distance class and extends all the way to the final distance class, in contrast to the male distances.

The median dispersal distances for males (median = 2095m) and females (median = 2479m) were not statistically different (*Z* = 1.27, *P* = 0.203, see Fig 2). However the distribution of the inverse cumulative proportions of dispersal distances into each distance class fit the exponential negative decay function very well (*R* ^2^ = 0.9898, *F*_(1,13)_ = 1168.19, *P*<0.0001). These distributions were significantly different for males (*R* ^2^ = 0.9955, *F*_(1,13)_ = 2651.03, *P*<0.0001) compared to that for females (*R* ^2^ = 0.0543, *F*_(1,13)_ = 0.6887, *P* = 0.4228), and showed no overlap in the 95% confidence intervals. Importantly, female dispersal distances did not fit the exponential decay distribution. The cumulative inverse proportion distribution for males reaches asymptote very rapidly (at short distances) compared to that for the females (Fig 3), which have a much more protracted distribution starting only in the second distance class and stretching all the way to the largest distance classes. This further points to the concentration of male dispersers at shorter distances. The Kolmogorov-Smirnov test also showed that male and female distributions of dispersal distances were different, being shorter in males (D = 0.6296, *P*_*MC*_<0.0001; Fig 2).

We also found that the distributions of distances for assigned pairs and unrelated individuals (the null expectation) were significantly different (D = 0.4037, *P*_*MC*_<0.0001), with assigned pairs having a lower median distance. This result suggests that the size of the study area is not responsible for all of the patterns of dispersal distances observed among assigned pairs, although dispersal could still have been limited by the size of the available habitat.

### Indirect estimation of gene dispersal distances

Population size estimates ranged from *N*_*e*_ *=* 86 (95% CI 58–156) for TNE, to *N*_*e*_ *=* 304 (95% CI 229–405) for SANE (Table 2). Values for LDNE were intermediate to those of the other two estimators (*N*_*e*_ *=* 261, 95% CI 228–303).

**Table 2:** Estimates of the effective dispersal distances based on calculation of Wright's neigborhood size *N*_*b*_ 438.52. The effective denisty *D*_*e*_ was calculated for a study area of 24km^2^. The mean-squared parent-offspring distance σ was calculated for each estimate of *D*_*e*_.

Estimator	*Ne (95%CI)*	*D*_*e*_ *(95%CI)*	4π*D*_*e*_ *(95%CI)* ^a^	σ *(95%CI)*Km
SANE^1^	304 (220–405)	12.66 (9.16–16.87)	159.09 (115.13–211.95)	1.66 (1.43–1.99)
LDNE^2^	261 (228–303)	10.87 (9.5–12.62)	136.59 (119.32–158.57)	1.79 (1.66–1.91)
TNE^3^	25 (18–32)	1.04 (0.75–1.33)	13.08 (9.42–16.74)	5.7 (5.11–6.82)

^1^ Sibship-analysis based estimate of effective size

^2^ Linkage-disequilibrium based estimate of effective size

^3^ Temporal Estimate of effective size

^***a***^ notation for Wright’s neighborhood size

**σ** the mean squared distance between the locations of parents and their offspring

The estimated average kinship coefficient was 0.0127 (95% CI = 0.0091–0.0143, *P* = 0.0012) and *b*_*log*_ was -0.0022 (95% CI = -0.0028–-0.0019, *P* = 0.0034). The neighborhood size *N*_*b*_ estimated from the kinship coefficient and *b*_*log*_ was 438.52m. We calculated σ for this neighborhood size based on the different *D*_*e*_ estimates (Table 2). The estimated effective dispersal or gene dispersal distance ranged from 1.6 km to 5.7 km depending on the estimator used.

## Discussion

We found a high rate of paternity assignments and restricted male dispersal distances–compared to females. We also found gene dispersal distances that correspond well with estimates obtained using parentage methods. Our study makes an important contribution to the understanding of avian dispersal ecology–especially due to our success in estimating dispersal distances in the spatial context of gene flow, which is not possible with mark-resight methods [16].

The categorical parentage assignment approach can be biased depending on genotyping error rates or on the number of heterozygous individuals in the sample. We estimated low error rates in our samples and hence do not believe genotyping error to be factor in our estimates. As the probability of correct assignments increase with the homozygosity of individual loci, some biases may be expected in assignment of populations with a high degree of heterozygosity [38]. The sampled population has a relatively high heterozygosity (0.81), but it is not possible to determine if and to what extent our assignments are influenced by the genetic diversity. Additionally, the presence of relatives could potentially bias the number of observed assignments [37]. In our study, the size of the study area may have increased the probability that full and half sibs were present, leading to some error in assignments. However, this problem is minimized when the true parent is also sampled. Although we sampled up to 32% of breeding males over two years in the population, our sampling protocol of spacing collections across different grid cells helped to ensure that full or half-sibs were not sampled in a non-random fashion. Analysis of the different proportions of pairwise relatedness among our samples indicated a relatively low proportion of highly related individuals–pairwise relatedness of individuals captured in the same net (0.13) was not significantly greater than that of individuals captured in different nets (0.10) (Athrey G, Barr K, Lance R and Leberg P, unpublished). Hence we do not expect a systematic error in our assignments arising due to the presence of multiple relatives within the sample. Even allowing for some error at the relaxed confidence level, using the assignments with high-confidence still provides a very informative view of black-capped vireo dispersal distances. The ad-hoc analysis of using assigned parents as ‘known’ fathers or mothers did not change the assignments in a substantial way, and especially did not increase the number of maternal assignments. The analysis with the known mother also did not increase paternal assignments or did not provide new information on extra-pair matings. This finding is not unexpected given the low number of females in our samples. However, as we do not have observation data on social pairs, it is impossible to determine if social pairings differ from genetic assignments of parentage. There were fewer assignments than expected at the strict confidence level whereas there was greater number of assignments than expected at the relaxed confidence, based on the initial simulation step. In the former case, this suggests that the dataset has the power to detect a greater number of true parent-offspring pairs (assignment power). In the latter (relaxed) assignment category, the greater number of observed assignments compared to expected suggests that there is some loss in assignment power at this confidence level. This is expected under the relaxed confidence criteria. The inferred dispersal rate based on assignment rate (64%) could be as high as 36%. While this rate is plausible, they are probably overestimates as observational and genetic estimates of dispersal suggest restricted dispersal [29]. Additionally, although we sampled about 20% of the potential parents in this population; it is quite likely that unsampled individuals parented some of the unassigned individuals.

Looking at the distribution of dispersal distances, more of the longer dispersal distances were associated with females and more of the shorter dispersal distances were associated with males, although the median distances were similar for both sexes (Figs 2 and 3). This pattern confirms the observations from our previous work showing restricted dispersal in males based on spatial autocorrelation, although we do not know the spatial range over which this remains consistent. Furthermore, the results showing that the female dispersal distances do not fit the exponential decay function likely have implications for management decisions. This result, taken together with the lower maternity assignments, suggests that females disperse much further than males, and in our study, over larger distances than the study area. Such a pattern can result in increasing genetic structure over time due to the fact that most measurable gene flow is due to the movement of one parent. If sex-biased dispersal results in sex-biased gene flow, then it greatly undermines the value of population structure estimates based on uniparentally-inherited markers (eg. mtDNA loci).

The complementary approaches of parentage assignment and estimation of gene dispersal based on the kinship coefficient provided a remarkably similar picture of the social and dispersal dynamics of black-capped vireos: both methods produced overlapping estimates of dispersal distances, indicating temporally proximate and evolutionary measures of gene flow to be occurring on similar spatial scales. Our study adds to the body of literature where similar approaches have been used in several recent studies to explore social structure, dispersal distances or genetic structure in different taxa. The main insights that our research has provided are in measuring and interpreting the consequences of dispersal two time-scales–the contemporary snapshot of dispersal based on parentage, and the evolutionary-time resolution of neighborhood sizes. Our work is also a step in an important direction for quantifying individual dispersal and gene dispersal in migratory birds, presenting a highly informative composite. Furthermore, few observational studies have the capability to conclusively link individual movement to measures of gene dispersal. Even when presumed parent-offspring movements are tracked, gene dispersal can be difficult to track as some offspring may result from extra-pair copulations. Thus, our study also emphasizes that broad applicability of parentage analysis methods for quantifying dispersal in various taxa, due to its much-reduced logistical footprint compared to direct observational methods.

### Paternity assignments

A large percentage of captured individuals in our dataset were assigned to fathers identified from the same population, indicating that males are philopatric and disperse to new territories within their natal population. This fits the general model for passerines where males are philopatric and females are the dispersing sex [60], and confirms our earlier finding based on spatial autocorrelation analysis [29]. Although these numbers can be indirectly inferred as the proportion of immigrating individuals, it is not possible to determine this in our present analysis. Telfer *et al*. [22] used a similar approach to infer the proportion of dispersing individuals, but in their case their data spanned both intra and inter-population samples and distances. Although we genotyped a large number of individuals, many of the apparent immigrants are likely to be parented within the population, but were not resolved as such due to incomplete sampling. Despite the incomplete sampling of possible parents, the relative difference in the immigration rates for males and females cannot easily be explained by insufficient sampling alone. There is no obvious reason why females should have lower relative rates of assignment than males due to under sampling of potential parents. This suggests that females are more likely than males to have originated from outside of the study population. Alternatively, it is possible that we were not able to determine dispersal distances for females due to the size of the study area itself. Although we sampled comprehensively in a relatively isolated population, our analysis did not include the nearest breeding population (110 km away). However, this may be difficult to evaluate without dramatically increasing the proportion of females sampled, and without other improvements in population genetic methods to identify long-distance migrants.

### Distribution of dispersal distances

One of our assumptions in inferring dispersal distances was that the capture location of assigned offspring was the territory occupied following natal dispersal, and we assumed no breeding dispersal. However, in black-capped vireos and other passerines, it is difficult to differentiate natal and breeding dispersal [61], specially when dispersal occurs within the natal population. Black-capped vireos are known to have high site fidelity and have been observed to return to the same nesting sites for multiple years [46, 62]. This suggests that the capture locations are their territories following natal dispersal. Hence dispersal distances estimated from assigned parent-offspring pairs are representative of natal dispersal distances.

We found that median dispersal distance in black-capped vireos was about 2.4 km from their putative natal sites. The distributions of dispersal distances were significantly different for males and females. While we had fewer female offspring assigned to fathers, proportions were standardized to sample size, and hence are not reflective of sampling error. It is difficult to directly compare these estimates to other estimates owing to the limited information on dispersal in this species. The furthest known distance that banded individuals traveled is 78 km [46]; there is little information on post-natal dispersal within populations. One source of potential error in the estimates of dispersal distances with this method is that it cannot account for the bias introduced into distance calculations due to extra-pair copulations. Extra-pair copulations are well documented in passerines [63–65]. In our study, as we did not track the putative parent-offspring distances by field observation, we do not know the frequency of extra-pair copulations and the impact it has on distance estimates. It is possible that some captured males were in territories that were not their own, and that some parent-offspring distance calculations are affected by this; hence we acknowledge that a small proportion of distance estimates could be inaccurate.

Our estimates of dispersal are within the range of observed natal dispersal distances in other birds [7], but they are at the lower extreme of the range. Paradis *et al*. [7] note that dispersal distances may be a function of several other factors that may influence dispersal distances, including population size, geographic range of the species, and whether the species is a resident or migratory species. However, it has to be noted that our study is among the very few that have quantified dispersal distances based on genetic analysis of parentage. It remains to be determined if the dispersal estimates from parentage analysis as reported here differ systematically from those obtained using observational approaches.

Generally, species with large population sizes, and positive population trends (increasing) are found to disperse shorter distances. However, migratory species are reported to have larger natal dispersal distances than resident birds [7]. The vireo population at KWMA was relatively small (approximately 400 singing males in an isolated habitat patch in 2007–08), but it has been increasing and the species is migratory, suggesting that dispersal distances should be large. However this prediction did not fit the data for black-capped vireos as most of our evidence of dispersal distances indicates restricted dispersal–at least in males [29]. Similar observations are emerging from other avian species [66, 67], and certainly from other vertebrates[68]. The assumption that a migratory species should have large natal dispersal distances deserves further examination. The relationship between the ability to travel long distances (for migration) and the propensity to disperse among breeding sites is not yet well resolved. Frequent and long distance dispersal may be crucial from a management standpoint for several threatened species to ensure genetic connectivity with other populations. Although our sampling method provided high resolution of dispersal behavior in black-capped vireos, our study also raises questions regarding temporal changes in this pattern or the consistency of this pattern across larger geographical areas. These considerations may be important for the black-capped vireo as it has suffered large fluctuations in available habitat and population sizes over the last century. Management of this species must consider the implications of variation in dispersal patterns resulting from differences in demographic and habitat conditions for effective conservation.

### Indirect estimates of gene dispersal

Estimating gene dispersal is crucial to understand the dynamics of populations in an evolutionary context. Using Wright’s equation for the estimation of neighborhood size is a valuable approach when direct observation is limited. However, Wrights equation for the estimation of neighborhood size is dependent on the accuracy of the underlying data–including the estimates of the effective density (*D*_*e*_) and the neighborhood size (*N*_*b*_). Due to the intensive data requirement to increase accuracy, a disproportionate number of studies are on plant species [48]. Recent improvements in estimators for the genetic effective size make this approach feasible on smaller datasets for other, mobile taxa.

Our estimates of *N*_*e*_ based on three different estimation approaches ranged from 25 to 304. Estimates of *N*_*e*_ can suggest different things based on the estimator used—for example differences in the coalescent effective size or the harmonic mean over an interval. Thus the estimates from the three methods employed here provide a range that captures the different demographic processes in this study population. The LDNE and SANE estimates were surprisingly similar, and given the large sample size used in this estimation, they may be considered accurate estimates. The TNE estimate was much smaller (25) but as this is a temporal estimate, this may represent the longer-term effective population size. The usual pitfalls of the temporal method apply in this case too, as this method is not expected to perform well in species with overlapping generations or when the number of generations separating samples is less than four [69].

Estimates of genetic neighborhood size ranged from 1.6 to 5.7 km based on the *N*_*e*_ estimates. Interestingly this range matches very well with distances for mean natal dispersal distances inferred from parentage assignment. This similarity of estimates obtained from independent methods provides support for the hypothesis that most dispersal in black-capped vireos occurs on a limited spatial scale.

It would be valuable to determine if the correspondence between the two approaches (parentage based and neighborhood size) are common in other systems and over a range of sample sizes, and geographic scales. While estimation of dispersal based on the neighborhood size is not as sample intensive as parentage assignment, it depends on other estimates to be unbiased for its accuracy. By using a variety of *N*_*e*_ estimators, we have included this source of variation in calculation of the effective density (*D*_*e*_). Although this range of *N*_*e*_ values results in differences in estimates of dispersal distances, the estimates all reflected limited dispersal.

Estimates of dispersal distance obtained from parentage assignment and effective dispersal, despite overlapping in this present study, represent individual and whole population parameters, respectively. Parentage assignment estimates are temporally proximate and are independent of the reproductive success of the dispersing individual. This is a critical indicator of demographic trends in the population, and potentially of value for management and conservation, as it is relevant to demographic connectivity and stability. On the other hand, effective dispersal is more relevant from an evolutionary perspective as it is a measure of individual gene dispersal distances, averaged at the population level, and potentially refers to genetic connectivity. Gene dispersal distances, due to its dependence of *F*-statistics based estimators, represents an average of dispersal occurring over several generations and would only incorporate dispersal that resulted in reproductive success (hence, effective dispersal) [47]. The convergence of estimated distances between these two approaches in our study may be a result of the high proportion of mating individuals sampled in this study, and the isolated nature of this population. Alternately, most dispersal in this system may result in reproduction. Another important suggestion from our study is that genetic connectivity may be poor in this species if the concordance of proximate and evolutionary measures of gene holds consistent across populations. This hypothesis needs to be further evaluated. In general, it would be important for future studies of avian dispersal to determine whether patterns observed in this study are consistent over time and space, or the extent to which they vary in response to environmental perturbations.

## Conclusions and Implications

Most importantly, we were able to quantify dispersal distances in male and female black-capped vireos–at a resolution exceeding that obtained from typical mark-recapture studies, and with comparatively limited logistical effort. We found new lines of evidence for sex-biased dispersal and limited dispersal distances using two complementary genetic approaches in the black-capped vireo. Males are found to disperse shorter distances than females. An extension of these approaches will be to provide a better understanding of temporal variations in dispersal rates, but also to determine the spatial scale over which these processes operate. Quantification of dispersal distances using the approaches presented here can also be beneficial in a variety of fields, such as understanding colonization and expansion of populations, tracking the movement of infectious diseases, and design of corridors for conservation.

Our results also have important repercussions for the management and conservation of black-capped vireos. Sex-biased dispersal has been suggested as a mechanism that increases population differentiation [70, 71]. As we have established in previous studies, fragmentation of black-capped vireo habitat has been associated with increased genetic differentiation and decreased within population genetic diversity in this species [14, 31]. With limited dispersal, management plans can succeed through the maintenance of early successional habitats within a few kilometers of other sites with breeding populations. Our study is another important contribution to the understanding of dispersal in neotropical migratory passerines, which have been much studied, but whose dispersal behaviors are not fully characterized [60]. Research in other species will be necessary to assess whether the correspondence between individual dispersal and gene flow observed in black-capped vireos holds for similar avian species.
